# Work-Life Imbalance: A Challenge and an Opportunity for Neurosurgery

**DOI:** 10.1227/neuprac.0000000000000140

**Published:** 2025-05-09

**Authors:** Sylvia Shitsama, Janissardhar Skulsampaopol, Ashirbani Saha, Michael D. Cusimano

**Affiliations:** * Department of Surgery, Division of Neurosurgery, University of Toronto, Toronto, Ontario, Canada;; ‡ Department of Surgery, School of Medicine, Jomo Kenyatta University of Agriculture and Technology, Juja, Kenya;; § Department of Surgery, Faculty of Medicine, Ramathibodi Hospital, Mahidol University, Bangkok, Thailand;; ‖ Department of Oncology, School of Biomedical Engineering, McMaster University, Hamilton, Ontario, Canada

**Keywords:** Neurosurgery, Work-life balance, Workload, Burnout, Workforce requirements

## Abstract

**BACKGROUND AND OBJECTIVES:**

Work-life balance (WLB) is the individual's view that personal and professional activities in their life align with current life priorities. WLB is important for health and is thought to prevent burnout in the workplace. Although high rates of burnout exist in neurosurgery (NS), studies of WLB and the factors that influence WLB in NS are not known.

**METHODS:**

An electronic international survey using a physician wellness framework was conducted globally. χ^2^ tests were used to analyze the association between WLB and age, sex, level of practice, and continent of practice.

**RESULTS:**

Of 446 respondents (65% staff, 35% trainees; median age range 35-44 years age category; 28% women), only 42% indicated the presence WLB. The presence of WLB was significantly lower in trainees compared with staff (χ^2^ = 14.065, *P* = .0002, odds ratio [OR]: 0.45 [95% CI: 0.30-0.68]), those aged 44 years and below (χ^2^ = 4.1464, *P* = .04172; OR: 0.63 [95% CI: 0.41-0.96]), and those in the African region compared with non-African region (χ^2^ = 8.33, *P* = .0039, OR: 0.42 [95% CI: 0.24-0.75]).

**CONCLUSION:**

Nearly two-thirds of those in NS report poor WLB with trainees and younger individuals at particular risk. Lack of sufficient numbers of neurosurgeons for the workload and the lack of support staff require urgent attention globally. There is an urgent need for healthcare organizations globally to take leadership in implementing practices to improve WLB. Evidence shows these changes will likely improve personal and organizational well-being, retention, and improve medical student interest in NS.

**ABBREVIATIONS:**
NS: neurosurgery
WLB: work-life balance.

Work-life balance (WLB) is defined as the state in which a person achieves equilibrium by equally prioritizing their career and personal life, minimizing conflict.^1^ In today's social landscape, satisfaction with WLB is fundamental for maintaining a healthy and motivated workforce.^2^ From a neurosurgical perspective, WLB involves balancing clinical and nonclinical responsibilities such as teaching, research, and life away from clinical duties.^3^

Neurosurgeons and neurosurgical trainees face substantial workload because of a global shortage of neurosurgical professionals, resulting in approximately 5 million unmet neurosurgical operations.^4^ This substantial difference between workload and a workforce capable to institute it is compounded by the acute needs of patients and surgeons' dedication to their patients' well-being. This leads to difficulties in establishing boundaries, which can affect their overall well-being and job satisfaction.^3,5-7^

Numerous factors significantly affect the WLB of neurosurgeons, including sex, residency status, and the environment they work in. Female neurosurgeons and trainees often grapple with conflicting responsibilities regarding career advancement and family commitments, exacerbated by insufficient support for maternity and childcare.^1,8^ Neurosurgical residents and their partners frequently voice concerns about heavy workloads impeding their personal lives, leading to dissatisfaction with their WLB.^1,8^

Interestingly, academic neurosurgeons in China report lower burnout rates and higher job satisfaction than their counterparts outside academia. They cite higher salaries, better career prospects, and safer working conditions in tertiary hospitals with training programs as key factors.^9^

In response to the negative effects of impaired WLB among neurosurgeons and neurosurgical trainees, institutions have implemented initiatives aimed at promoting WLB. These include a renewed focus on mentorship, wellness, programs, and team-building activities.^5^ None of these initiatives seek to reduce actual workload.

Neurosurgeons and their trainees continue to struggle with WLB because of the persistent belief that it is a personal issue rather than one influenced by the surrounding environment. As a consequence, as wellness initiatives tend to be designed at the organizational level, they often overlook individual factors within specific contexts including but not limited to sex, training environment, type of practice, individual perceptions, and the potential solutions to address these concerns. Furthermore, solutions aimed at reforming or changing the actual work environment and culture of an organization/institution are challenging to implement.^10^

Although multiple initiatives have been implemented at the organizational level, little effort has been made to understand WLB at an individual level and create context-specific solutions.

This study sought to understand the perception of WLB among neurosurgeons and trainees based on age groups, sex, level of practice, type of practice, and geographical location. The study also explored organizational policies put in place to enhance WLB while examining barriers and proposed solutions.

## METHODS

This study involved an electronic survey (**Supplemental Digital Content 1** [http://links.lww.com/NS9/A43]) based on a physician wellness framework to examine the presence of WLB, identify perceived barriers, and propose potential solutions. The survey was circulated to neurosurgeons and neurosurgical trainees (residents and fellows) across continents through email and social media platforms, in collaboration with various neurosurgical organizations.

A combined WLB score was derived from responses to 4 specific questions (Table 1) identified by the team on 4 items that are related to job satisfaction, quality time, workload, and WLB. For computing this composite score, we recorded the levels (of the answers to the 4 questions) Strongly Disagree, Disagree, Neither Agree nor Disagree, Agree, and Strongly Agree, respectively, to 1, 2, 3, 4, and 5 for all the positive questions (questions that ask about positive effect of WLB). For any negative question (only 1 such question was included), we assigned -1, -2, -3, -4, and -5 to Strongly Disagree, Disagree, Neither Agree nor Disagree, Agree, and Strongly Agree, respectively. Therefore, the composite WLB score lies between -2 (derived from 4 questions as 3*1-5) and 14 (= 3*5-1). Based on this range, the mid-value of this score is 6. Furthermore, the median value of the composite score is also 6. We derived a binary version of the score to indicate the presence of WLB (score value >6) and absence of WLB (score value ≤ 6). Descriptive statistics, χ^2^ tests, and odds ratio were used to explore the relationship between WLB (whether present or absent) and factors such as age, sex, and type and level of practice, with a significance level set at *P* < .05. Qualitative analysis was conducted using the thematic analysis methodology.

**TABLE 1.** The 4 Questions Used to Develop the Work-Life Balance Composite Score

Q7.1: I have enough time to perform both of my work and personal life duties
Q31.3: I have adequate time for my family
Q31.10: I am content with the balance between my work and home life
Q29.6: I have difficulty balancing my professional and personal life

The study was approved by the Unity Health Toronto Research ethics board REB# 20 -323 and consent form appended on the first page of the survey link.

## RESULTS

### Demographics

Of 659 participants, 446 (68% completion rate) provided complete answers to the 4 questions used to derive the WLB composite score. Of the respondents, 70% were male, 28% female. 2% preferred not to answer the related question (Table 2). Among the respondents, 65% were neurosurgeons, and 35% were trainees with a median age corresponding to the age category of 35-44 years.

**TABLE 2.** Presence of WLB Based on Sex, Age, and Type of Practice

Work-life balance	Absence (N = 258)	Presence (N = 188)	*P* value
Sex			
Male	55%	45%	.1
Female	64%	36%	
Age			
≤44 y	61%	39%	.04
>44 y	50%	50%	
Type of practice			
Academia only	56%	44%	.1
Both academia and nonacademia	67%	33%	
Nonacademia	55%	45%	

Of the participants, 42% reported the presence of WLB with a slightly lower rate among women compared with men (36% women, 45% men).

The presence of WLB was significantly lower in participants aged 44 years and below (χ^2^ = 4.1464, df = 1, *P* = .04; odds ratio [OR]: 0.63 [95% CI: 0.41-0.96]) compared with those aged 45 years and older.

The presence of WLB in participants with combined work practice in academia and nonacademia was lower (33%) than in academia (44%) or nonacademia (45%) alone.

**FIGURE 1.**
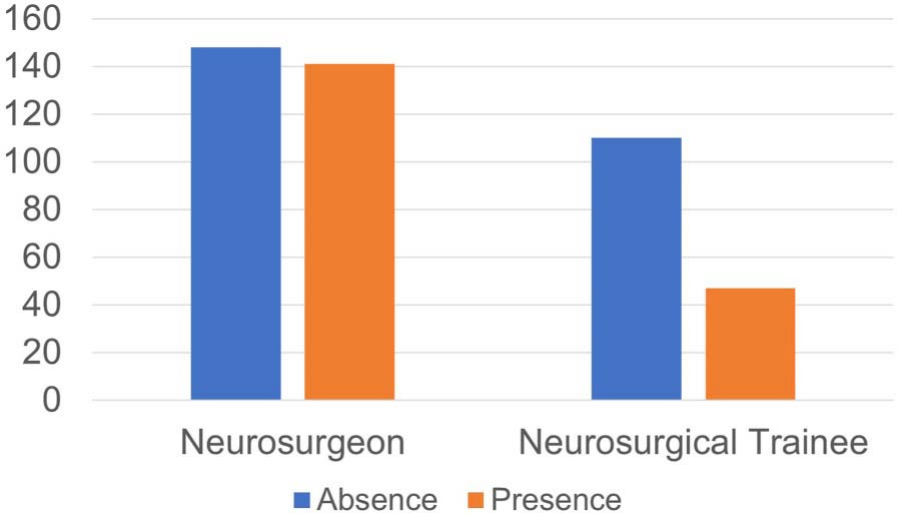
Respondents with the presence and absence of WLB according to level of practice. The presence of WLB was significantly lower among the neurosurgical trainees compared with neurosurgeons (*P* = .0002, odds ratio: 0.45 [95% CI: 0.30-0.68]). WLB, work-life balance.

The presence of WLB was significantly lower among the neurosurgical trainees compared with neurosurgeons (χ^2^ = 14.065, df = 1, *P* = .0002, OR: 0.45 [95% CI: 0.30-0.68]) (Figure 1).

**FIGURE 2.**
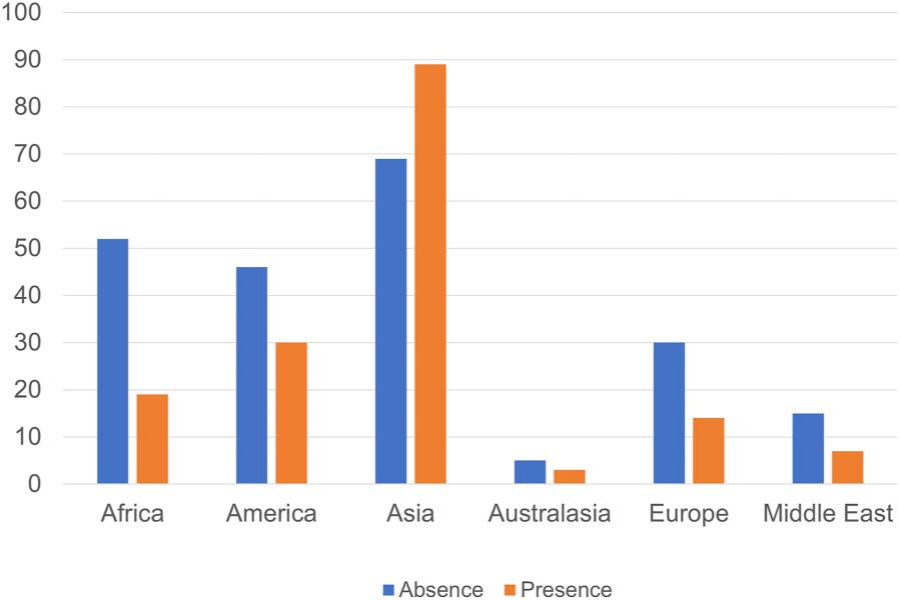
Respondents with the presence and absence of WLB according to the geographical region. The presence of WLB was significantly lower among respondents from African region compared with those from non-African region (*P* = .0039, odds ratio: 0.42 [95% CI: 0.24-0.75]). WLB, work-life balance.

The presence of WLB was significantly lower among respondents from African region compared with those from non-African region (χ^2^ = 8.33, df = 1, *P* = .0039, OR: 0.42 [95% CI: 0.24-0.75]) (Figure 2).

### Barriers to WLB

Four broad themes emerged from the analysis of barriers to WLB: They all focused on very heavy work demands related to Workforce supply, financial needs leading to further working hours, and tension between work and family responsibilities.

- Staff shortage
  Participants emphasized insufficient staffing in relation with the work demands put on neurosurgeons, leading to long work hours that subsequently had an impact on their work hours.
  One male neurosurgical trainee said…
  Neurosurgery is SEVERELY understaffed, too much work to be shared by few
- Heavy workload
  Several participants voiced concerns that inadequate staffing, coupled with inefficient work practices, referral processes, and high patient acuity, resulted in significant workload demands on neurosurgeons and trainees.
  One male neurosurgeon said….
  Heavy call days, disorganized work at the hospital and many unnecessary referrals from other hospitals and the lack of time at work to perform the scientific and educational activities
- Financial needs
  Some participants felt compelled by unpaid programs to take on further workload to cover their tuition and living expenses, resulting in limited personal time.
  One male neurosurgical trainee said …
  Lack of pay during residency means I have to use some of my off-duty time to do locum duties in a private hospital to earn some little money to keep me going and if possible cover some of the heavy tuition fees. That leaves me with very little time for myself. I have very little time for socializing, or for even starting a family of my own.
  Moreover, neurosurgeons perceived the lengthy training shortens their lifetime earning period, prompting them to work longer hours to meet financial obligations.
  One male neurosurgeon said…
  Late start earning/short window to save for retirement means I have to keep long hours in order to provide for family and hope to retire.
- Family responsibilities
  Heavy workload coupled with inadequate staffing resulted in long working hours affecting time spent with family.
  One female neurosurgical trainee said…
  It is difficult to have sufficient time to spend with children when you work 2 out of 4 weekends for 6 years.

### Proposed Solutions to Improve WLB

Most participants emphasized the importance of addressing the mismatch between workforce supply and demand. The disparity between the number of neurosurgeons and the support staff needed to meet the growing demand for neurosurgical services was particularly a salient theme. Less than one-third of participants indicated that their organizations provided support for WLB, with flexible work hours being the most commonly available benefit. Moreover, 2 key strategies emerged as potential solutions for improving WLB: fostering personal initiatives and encouraging a shift in organizational culture.

- Workload management
  A number of the participants felt that increasing the workforce through training and hiring coupled with task reallocation of administrative duties to support staff would ease the workload burden.
  One male neurosurgeon said…
  Get more physician assistants, nurse practitioners and clinical associates to assist with day-to-day duties. Have a minimum of 3 surgeons working together to provide some level of care for a problem.
- Personal Initiatives
  Some participants suggested that individuals should take greater personal responsibility for achieving a balance between work and family commitments, which could be performed by establishing clearer boundaries and prioritizing family time as sacred.
  One female neurosurgeon said…
  Do not take work with you outside the work place
  One male neurosurgeon said…
  It is awareness and respecting the “whole person's work life” Being aware of the importance of WLB in productivity, skill development and maintenance, and the “joy” of and at work and beyond
- Organizational Culture Shift
  Participants indicated that organizations should develop programs aimed at promoting a balance between work and personal life. These programs could encompass various measures including accommodating flexible work hours, fair compensation relative to the workload, providing childcare services designed to accommodate the demanding schedules typical in neurosurgery (NS), and offering mentorship opportunities.
  One female neurosurgical trainee said…
  Increase payment, flexible working hours

## DISCUSSION

### Key Results

This international study evaluated the presence of WLB among neurosurgeons and their trainees, and barriers to achieving this balance and proposed potential solutions. Our findings show that low levels of WLB in NS are seen in every region across the world. This disparity is particularly evident among trainees and neurosurgeons aged 44 years and younger, as well as in those practicing within the African region. Our results echo previous research indicating a continued low level of WLB in NS and are similar to those of comparable studies conducted among Korean and European neurosurgeons.^11,12^

Several factors may contribute to these findings in younger neurosurgeons. During training, residents face substantial workloads and frequent rotations, leading them to prioritize work over personal time to meet training requirements.^13-15^ This persistent poor WLB has been linked to hindered learning, increased burnout, and impaired well-being.^16^ Training organizations must conduct a comprehensive evaluation of the workload assigned to trainees and develop strategies to redistribute tasks to other members of the healthcare team, such as clerical staff, physician assistants, or nurse practitioners. This reallocation should be implemented in a manner that preserves the educational opportunities for trainees.

Our results also show that despite strides toward inclusivity in NS, sex-specific challenges persist. Consistent with the existing literature, this study reveals that women experience lower levels of WLB compared with men. This discrepancy may stem from the child-bearing factors that women face and the absence of policies supporting childbearing and inflexible training programs with rigid work schedules.^11^ These challenges, in turn, affect job satisfaction, WLB, and retention of female neurosurgeons in the workforce. Multifaceted solutions such as flexible working schedules, mentorship, and policy changes at an organizational level to foster a supportive work environment will be required to attract and retain women in NS.^11,17^

Our analysis indicates that heavy workloads in NS are primarily driven by a shortage of staff and the high demands placed on existing personnel. Participants in our study identified staff shortages and excessive workloads as key factors negatively affecting WLB. These findings align with previous research, which has shown that substantial workloads and rigid schedules limit personal time, contributing to burnout, job dissatisfaction, and higher attrition rates.^12,18^ It is crucial to recognize that healthcare systems worldwide are experiencing a significant shortage of personnel to manage neurosurgical patients, which represents both a substantial challenge and an opportunity for future advancement. An immediate and comprehensive re-evaluation of workforce projections is necessary, as the number of neurosurgeons required to meet clinical demands must consider factors such as demographic aging, increasing patient complexity, and the evolving expectations of incoming professionals.^19^

Efforts to address this shortage must include scaling up training programs to accelerate workforce growth through coordinated actions by governments, neurosurgical societies, institutions, and philanthropic organizations,^20^ with these efforts being integrated into broader health system planning. Future workforce models should also incorporate considerations such as projected career duration, flexible work hours, and shared full-time positions that foster professional well-being. The current status quo is unsustainable, and failure to adapt workforce projections to these factors will exacerbate shortages, ultimately undermining the ability to meet global patient care needs.

Regarding expectations of future generations in NS, a recent Canadian survey on physician work hours across all specialties has shown a notable reduction in hours worked, indicating a potential prioritization of enhanced WLB among physicians.^21^ Failure to meet these evolving expectations in the future, coupled with an insufficient number of neurosurgeons to manage the demands of their role, may further exacerbate existing imbalances of WLB. Addressing the issues that impair WLB have the benefits of better mental health and increased productivity.^22^

Our findings suggest that without increasing the workforce and redistributing tasks, the existing unmet needs—evidenced by long waiting lists and limited access to neurosurgical services—will likely worsen. This presents both a significant challenge and a valuable opportunity to reform NS training and work environments. Those responsible for funding and delivering neurosurgical services must take immediate action to address workforce shortages and the workload expectations placed on neurosurgeons. It is crucial to develop policies that support lifelong learning and create working conditions that address the specific needs of both women and men in NS.^23^

To achieve WLB at an individual level, it is crucial to place the employee at the center of policy development and implementation. This ensures the creation of practical and effective solutions, which should involve setting clear priorities, managing time effectively, and establishing realistic goals.

### Limitations

There are certain limitations to our study. As a worldwide survey, there is the potential of selection and response bias as some countries had fewer respondents; however, we had diverse representation from all geographic areas. Our results may also indicate a lower bound of the WLB problem in NS because our nonrespondents may have higher rates of dissatisfaction with their WLB than those who replied. Despite these possible limitations, the current data provide sufficient insight into the general WLB within the neurosurgical community globally to institute action to address these important findings.

## CONCLUSION

We found that the neurosurgical community struggles with a poor level of WLB that particularly affects trainees and younger neurosurgeons. Heavy and sometimes inappropriate workloads, compounded by staff shortages and prolonged, unpaid training programs, are exacerbating this situation adding financial pressures. There is need to increase workforce numbers of neurosurgeons and staff to support those neurosurgeons globally to meet the massive demand for neurosurgical services in every international jurisdiction. Addressing these issues requires new workforce studies that address evolving expectations of WLB and multifaceted approaches at both the individual, organizational level, and country levels to promote WLB. The insights gleaned here can guide policy development to improve WLB. Organized NS must lead the development of policies that incorporate the diverse voices of young neurosurgeons everywhere in a way that addresses WLB and, at the same time, meets the needs of the populations of patients globally. Meeting this challenge with urgency will have positive effects on neurosurgeons and the patients they serve.

### Generalizability

The global distribution of the survey allowed for the representation of participants from all continents, therefore allowing for the generalizability of the results.

## SUPPLEMENTAL DIGITAL CONTENT

**Supplemental Digital Content 1**. Questions completed by the participants.
